# Temperature and Photoperiod Interactions with Phosphorus-Limited Growth and Competition of Two Diatoms

**DOI:** 10.1371/journal.pone.0102367

**Published:** 2014-07-10

**Authors:** Tom Shatwell, Jan Köhler, Andreas Nicklisch

**Affiliations:** 1 Department of Ecohydrology, Leibniz-Institute of Freshwater Ecology and Inland Fisheries (IGB), Berlin, Germany; 2 Department of Ecosystem Research, Leibniz-Institute of Freshwater Ecology and Inland Fisheries (IGB), Berlin, Germany; University of Connecticut, United States of America

## Abstract

In lakes, trophic change and climate change shift the relationship between nutrients and physical factors, like temperature and photoperiod, and interactions between these factors should affect the growth of phytoplankton species differently. We therefore determined the relationship between P-limited specific growth rates and P-quota (biovolume basis) of *Stephanodiscus minutulus* and *Nitzschia acicularis* (diatoms) at or near light saturation in axenic, semi-continuous culture at 10, 15 and 20 °C and at 6, 9 and 12 h d^−1^ photoperiod. Photoperiod treatments were performed at constant daily light exposure to allow comparison. Under these conditions, we also performed competition experiments and estimated relative P-uptake rates of the species. Temperature strongly affected P-limited growth rates and relative P uptake rates, whereas photoperiod only affected maximum growth rates. *S. minutulus* used internal P more efficiently than *N. acicularis*. *N. acicularis* was the superior competitor for P due to a higher relative uptake rate and its superiority increased with increasing temperature and photoperiod. *S. minutulus* conformed to the Droop relationship but *N. acicularis* did not. A model with a temperature-dependent normalised half-saturation coefficient adequately described the factor interactions of both species. The temperature dependence of the quota model reflected each species’ specific adaptation to its ecological niche. The results demonstrate that increases in temperature or photoperiod can partially compensate for a decrease in P-quota under moderately limiting conditions, like during spring in temperate lakes. Thus warming may counteract de-eutrophication to some degree and a relative shift in growth factors can influence the phytoplankton species composition.

## Introduction

Interactions between nutrients and physical factors like temperature and light are important for phytoplankton growth. Nutrients and light are interdependent in surface waters because low nutrient levels restrict the phytoplankton biomass, and therefore typically coincide with clearer water. In turn, clearer water leads to higher underwater irradiances [1] and a longer effective photoperiod due to a deeper euphotic depth [2]. Turbidity on the other hand increases absorbed radiation and therefore influences the water temperature and thermal structure [3], [4], and water temperature affects nutrient cycling processes [5]. Therefore, in addition to seasonal cycles, the relationships between nutrients and physical factors can change, for example, due to global warming or eutrophication [5], [6], with consequential effects on phytoplankton communities that can be difficult to predict. For example, the dominance of filamentous cyanobacteria during spring in a shallow lake depended on the combined effects of winter temperature and the P:Si ratio [2] and the timing of the phytoplankton bloom was synergistically affected by water temperature and phosphorus supply [6]. The occurrence of several *Aulacoseira* (formerly *Melosira*) species could be differentiated along an irradiance/photoperiod – phosphorus gradient, suggesting trade-offs between these factors [7]. Understanding these effects may depend on how well we understand the physiological response of individual species.

While Liebig’s Law of the Minimum is assumed to apply to interactions between nutrients, this is not the case between physical factors like temperature, light and the photoperiod [8], or between nutrients and physical factors [9]. Temperature and photoperiod affect maximum specific growth rates of phytoplankton [10] and were shown to have species-specific interactive effects on nutrient-replete growth, where for example the response to changes in the photoperiod under constant daily light exposure depended on temperature [11]. However, these interactions may be different under nutrient limitation because temperature and light not only affect growth rates, but also N- and P-quotas [12]–[14]. At the same time, light and temperature also influence nutrient uptake rates in a nutrient- and species-specific manner [15], whereby the temperature dependence of uptake is typically different to that of growth [16].

While the photoperiod was shown to have an important effect on spring phytoplankton [11], its interaction with phosphorus limitation is relatively unexplored. If phosphorus uptake rates differ in the dark and light [17], then there may be an interaction between phosphorus and the photoperiod [18], particularly given the influence of light on phytoplankton stoichiometry [19]. Litchman *et al*. [20] showed that the combined effects of photoperiod and P-limitation were species-specific and greater than the sum of individual effects for several phytoplankton species, whereas Riegman and Mur [21] found a more either/or type of limitation between photoperiod and phosphorus for *Planktothrix* (formerly *Oscillatoria*) *agardhii*. Overall, it seems difficult to form a conclusive picture from these studies on photoperiod interactions with phosphorus, also because comparison is difficult when the irradiance and not the daily light exposure is held constant in daylength treatments. Under nutrient-replete conditions, light-saturated growth rates do not increase linearly with the photoperiod but show saturation characteristics [8], [11], [22]–[25]. Because of this nonlinear response, the effect of the photoperiod on growth rates can only be compared if the daily light supply is kept constant.

Interactions between phosphorus and physical factors have become particularly relevant because recent research has stressed the importance of developing a mechanistic or biochemical basis for Droop’s [26] quota model, which successfully describes phytoplankton growth rates, stoichiometry and the dynamics of limiting and non-limiting nutrients [27], [28]. Droop’s model [*µ* = *µ*’_m_(1–Q_0_/Q)] relates the growth rate (*µ*) to the nutrient quota (Q) in terms of the minimum quota (Q_0_) and the theoretical maximum growth rate at infinite quota (*µ*’_m_). Q_0_ is the quota at which growth is zero and represents the amount of nutrient required for cell structure and machinery [28]. Q_m_ is the maximum quota at the real maximum growth rate when the nutrient is not limiting. The quota can exceed Q_m_ under luxury consumption, but this excess P is essentially stored as polyphosphate and does not allow an immediate further increase of the growth rate [29]. The form of Droop’s quota curve is fixed by the ratio Q_0_:Q_m_, or expressed differently, such that the curve is half-saturated when the quota is double Q_0_. Q_0_ seems to decrease with increasing temperature [13], [30]–[32], although there are exceptions [33], [34]. Furthermore, not only the maximum growth rate and Q, but also Q_0_:Q_m_ [30] are all temperature dependent. Because the upper part of the quota curve is probably more important in deducing competitive advantage between species than the lower part near Q_0_ [35], the Droop model may not be sufficient in characterising temperature or photoperiod interactions with P-limited growth. Of equal if not greater importance than the growth-quota relationship are the nutrient uptake kinetics, and the feedback between uptake and quota [27]. The growth-quota relationship thus needs to be considered in conjunction with uptake.

In this study we therefore investigate the effects of temperature and photoperiod on phosphorus-limited growth by comparing the parameters of the P-quota curve fitted to P-limited growth rates of two diatoms measured in semi-continuous culture under different temperatures and photoperiods. Due to the typical transition from limitation by physical factors to nutrient limitation during the course of spring, we assume interactions may play an important role here and accordingly, we chose two spring blooming diatoms as test species. We also examine the shape of the quota curve and the applicability of Droop’s relationship. To enable comparison between photoperiod treatments, we adjusted the irradiance in experiments to compensate for different photoperiods and maintain a constant daily light supply [11], [36]–[39]. Predicting the outcome of competition under nutrient limitation depends largely on obtaining accurate uptake measurements, which can be difficult [40], [41]. As an alternative, we performed competition experiments to deduce the relative uptake affinity of the two species based on rates of competitive exclusion and the measured growth-quota kinetics. According to Healey [42], we assume that uptake affinity and competitive ability are proportional to the initial slope of the Michaelis-Menten uptake curve. Finally we extend an existing model that describes temperature-photoperiod-light interactions under nutrient replete conditions [11] to include phosphorus limitation. Our hypothesis is that both temperature and photoperiod influence the form of the phosphorus quota curve as well as the share of phosphorus uptaken by each species in competition experiments.

## Methods

### Ethics statement

No specific permissions were required to isolate the diatom strains used in this study at the given location. All samples were taken from public waters. No field studies were performed involving endangered or protected species.

### Algal strains

Growth experiments were performed under laboratory conditions for *Stephanodiscus minutulus* (Kütz.) Cleve and Möller (Bacillariophyceae), strain Mue0511A6, isolated in 2005 by Nicklisch, and *Nitzschia acicularis* W. Smith (Bacillariophyceae), strain Mue070319C1, isolated by Nicklisch in 2007. All strains were isolated from Lake Müggelsee (Berlin, 52.44°N 13.65°E) and were axenic.

### Algae Cultivation

*S. minutulus* and *N. acicularis* were grown under P limitation in semi-continuous culture according to the chemostat principle [43] and under P-replete conditions according to the turbidostat principle. Algae were cultivated in FW04 medium, a fully synthetic freshwater nutrient solution according to Nicklisch *et al*. [11] with an ionic-composition similar to the water of Lake Müggelsee, supplemented with a trace element solution according to Nicklisch [43] and a vitamin solution according to Guillard & Lorenzen [44] slightly modified (final concentrations: 1 *µ*g L^−1^ biotin, 1 *µ*g L^−1^ cobalamin and 100 *µ*g L^−1^ thiamine). The nutrients (300 *µ*M Si, 200 *µ*M N, 10 *µ*M P, 2 *µ*M Fe) were not limiting in the nutrient replete experiments at the low algal biomass densities we used. For P-limitation experiments, the P-concentration was decreased to 1.2 *µ*M P. The solution was allowed to equilibrate with air by shaking to get a pH of about 8.3 at 20°C and then sterilised by filtering it through a membrane of 0.2 *µ*m pore diameter. The solution was subsequently heated by microwave to just below 100°C but not allowed to boil.

Algae were cultivated in Erlenmeyer flasks of 300 mL filled with 100 mL suspension on an orbital shaker (50–90 rpm) in an incubator at 10, 15 and 20°C (±0.5°C). Irradiance was supplied from the side by fluorescent tubes of light colour Biolux and Warm White (Osram, Munich, Germany) at a ratio of 1∶1 and cultures were positioned relative to the light source such that each culture received the same irradiance. The scalar photon flux density of the photosynthetically available radiation (PAR in *µ*mol quanta m^−2^ s^−1^) was measured using a spherical sensor (QSL-101, Biospherical Instruments, California, USA). The light exposure in mol quanta m^−2^ d^−1^ was calculated as the sum of the irradiance over the photoperiod. Self-shading was minimised by the low biomass concentration (<300 *µ*g Chl *a* L^−1^) and the shallow depth of the culture suspension in the flasks.

### P-limited growth and competition experiments

In P-limited growth experiments, cultures were grown at 10, 15 and 20 °C with a photoperiod of 12 h d^−1^ to investigate temperature interactions, and under photoperiods of 6, 9 and 12 h d^−1^ at 15°C to investigate the photoperiod interactions. In the text, we use the term irradiance to refer to the instantaneous photon flux density, and the term light exposure to refer to the total daily light supply, often called the daily dose. We avoid the use of dose because this is *sensu stricto* absorbed light [11], [45]. Light exposures were saturating or near saturating for all experiments (all growth rates were ≥80% of the asymptotic maximum growth rates) as determined from growth-irradiance curves for these species measured at the experimental temperatures and photoperiods [11], [36], [38]. Irradiances in the experiments were adjusted to maintain a roughly constant daily light exposure of 3–5.6 mol m^−2^ d^−1^. First, nutrient replete cultures were acclimated to the desired conditions of temperature, photoperiod and irradiance for 5 to 7 days. During this phase and the following experiment, the biomass and growth rates were determined every day or every second day (at low growth rates) as described below. After acclimation when the cultures had reached balanced growth, the maximum specific growth rates were determined in turbidostat mode. Here cultures are diluted to a constant starting biomass each day and the dilution rate is adjusted accordingly. The growth rate is then determined from the dilution rate. The growth experiments were carried out for two to four weeks to get a mean of the specific growth rate. The longer measuring periods were needed for low growth rates at low temperatures or short photoperiods.

To determine P-limited growth rates, nutrient replete cultures, which had been acclimated to the experimental conditions as described above, were diluted with P-free medium in such a proportion to achieve a final concentration of 1.2 *µ*M P and subsequently allowed to starve for 2 to 5 days. The algae were then further cultivated semi-continuously with a nutrient solution containing 1.2 *µ*M P at a constant dilution rate (0.2–0.8 d^−1^). The cultures reached a steady state within 1 to 2 weeks so that the specific growth rate was equal to the dilution rate and biomass was constant at each dilution. It was assumed that the P added at dilution is absorbed very rapidly and that biovolume then increases practically linearly. The P-quota associated with the measured growth rate can then be calculated as the total P concentration per mean biovolume between dilutions. Here the actual short term growth rates measured between dilutions, which usually differed slightly from the applied dilution rates, were considered. The maximum yield (reciprocal of the minimum quota Q_0_) was determined by allowing the cultures to grow without dilution until they reached a maximum biomass (usually 1–2 weeks). The total P-content of cultures was checked at the end of the experiments by standard methods [46].

In competition experiments, axenic, unialgal cultures of *S. minutulus* and *N. acicularis* were first acclimated to the desired conditions and then grown under P-limitation at the dilution rate required for the later competition experiments. Once the cultures had reached balanced growth within 1–2 weeks, equal culture volumes of the two species were mixed so that each species initially contained 50% of the total phosphorus. Occasionally different proportions were used. The mixed cultures were further cultivated semi-continuously for one to three weeks at a constant dilution rate of 0.4–0.5 d^−1^ at 10, 15 and 20°C with a 12 h d^−1^ photoperiod and also at 15°C with a 6 h d^−1^ photoperiod. The proportions of each species were measured in fixed samples (0.25% glutaraldehyde final concentration) every one or two days using a flow cytometer (FACStar-Plus, Becton Dickinson, San Jose, USA) according to Nicklisch and Steinberg [47]. While there was some error in the absolute cell counts by the flow cytometer, the proportions of the species were accurate as verified by microscopic counts. Cells from the different species in the mixed cultures were distinguished based on their forward scatter signals and the Chl *a*-autofluorescence excited by a blue laser at 488 nm. In competition experiments, the competitive ability (CA) of the weaker species (*S. minutulus*) relative to the stronger species (*N. acicularis*) was quantified by
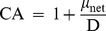
(1)where *µ*_net_ is the net specific growth rate (≤0) and D is the applied dilution rate. The competitive ability is thus 1 when the two species coexist and 0 when the weaker species does not grow and is washed out at the dilution rate.

### Biomass and specific growth rate measurements

During experiments, cultures were sampled every one or two days, always 3 hours from the beginning of the photoperiod and the biomass was determined by photometry at a wavelength of 436 nm (5 cm cuvette, Shimadzu photometer type UV-2401 PC). The focus of the light beam and the distance between cuvette and photomultiplier in this photometer excludes most of the scattered light, including forward scattered light, from detection. Therefore, the measured absorbance was caused by scattering (about 80%) and pigment absorption (about 20%). In parallel, chlorophyll fluorescence (F_o_) and variable fluorescence (F_v_) were measured using a Xenon-PAM Fluorometer (Heinz Walz GmbH, Effeltrich, Germany) after dark adaptation for at least 20 minutes. F_o_ is closely correlated to chlorophyll *a* content and F_v_, which is the increase in fluorescence above F_o_ after a light saturation pulse (Kautsky effect), is closely related to total PS II activity [48]. The chlorophyll and variable fluorescence were used to monitor the condition of the cultures and also as surrogates for biomass.

Specific growth rates were thus calculated separately using the change in absorbance at 436 nm, F_o_ and F_v_. The growth rates calculated with these three parameters are equal when the culture is in quasi-steady state. The three growth rate values (average difference 1%) were averaged to obtain the most accurate estimate of the true rate. Absorbance was used rather than direct biovolume measurements due to higher repeatability and shorter measuring and dilution times, which minimised culture disturbance. Before taking samples, we performed absorbance and fluorescence measurements to ensure that growth was balanced, in which case cultures are in quasi steady state. Here the biomass composition is nearly constant and the specific growth rates are not only biomass-specific but also C- or Chl *a*-specific, provided that the cultures are measured at the same time within the photoperiod.

### Phosphorus, carbon and chlorophyll content

The phosphorus content of cells was determined as the total phosphorus concentration in the fresh medium divided by the biovolume concentration of the culture at sampling, assuming that, under P-limitation and axenic conditions, all phosphorus was incorporated into the cells. Biovolume was measured with a cell counter (CASY, Modell TTC, Schärfe System, Germany). Carbon content was measured with a C/N analyser (Vario EL, Elementar Analysensysteme, Hanau, Germany) after collecting cells on pre-rinsed, pre-fired and pre-weighed filters (Munktell 25 mm). Chlorophyll *a* was measured for each culture at each dilution rate and experimental treatment with a Waters Alliance (Milford, USA) high performance liquid chromatography (HPLC) system with photodiode array detector, according to the method of Shatwell et al. [38]. In addition, each time a culture was sampled, chlorophyll content was measured using the chlorophyll fluorescence, F_o_, calibrated against the HPLC measurements. Chlorophyll content for each culture was then taken as the mean of the HPLC and fluorescence measurements.

### Statistics and model fitting

To analyse P-limited growth measurements, we fitted and compared different types of rectangular hyperbolic (HYP) and exponential (EXP) saturation curves, which describe the specific growth rate (*µ*) under P-limitation as a function of P-quota (Q):
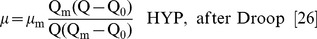
(2)




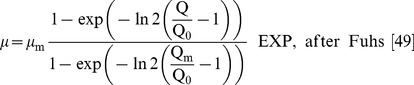
(3)


Here Q_0_ is the subsistence or minimum cell quota. The original Droop and Fuhs models were formulated in terms of the theoretical maximum growth rate at infinite quota (*µ*’_m_) but here have been reformulated using the real maximum specific growth rate (*µ*_m_) which occurs at the maximum quota (Q_m_).

In addition, we also fitted normalised quota models which contain a dimensionless shape parameter (*KQ, κ*) in place of a half saturation constant:
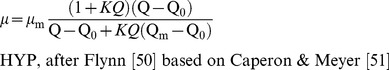
(4)

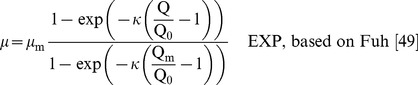
(5)The normalised shape parameters are related to the half-saturation constant (k_Q_) as follows:
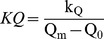
(6)




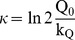
(7)


Since the Droop and Fuhs models are half saturated at 2Q_0_ (i.e. k_Q_ = Q_0_), the Droop relation applies when *KQ* = Q_0_/(Q_m_ – Q_0_) (Eq. 6) or when *κ* = ln 2 (Eq. 7). Accordingly in these cases, Eq. 4 becomes equivalent to Eq. 2 and Eq. 5 becomes equivalent to Eq. 3. *KQ* and *κ* are not directly comparable with each other and they should not be confused with k_Q_, which has the same units as Q. *KQ* and *κ* do however provide a basis for generalising the form of the quota curve irrespective of the units of biomass used [27]. Decreasing *KQ* and increasing *κ* (at constant *µ*_m_) indicate an increase in efficiency of P usage.

To analyse the effect of temperature and photoperiod on P-limited growth, we fitted Eq. 5 to each experimental treatment and examined the effect of temperature and photoperiod on the fitted model parameters. To determine whether the temperature or photoperiod effect was significant, we first fitted Eq. 5 to the pooled data from all temperature (or photoperiod) treatments. We then added a linear temperature (or photoperiod) dependence to the model parameters and refitted the model. These more complex models were subsequently compared with the simpler model using F-tests according to Bates and Watts [52]. If the residual variance of the more complex model with the temperature (or photoperiod) dependence was significantly lower than the residual variance of the simpler model without a temperature (or photoperiod) dependence (after accounting for the increased model complexity), we inferred that the temperature (or photoperiod) effects were significant. Eqs 2–5 were compared with Akaike’s Information Criterion (AIC), which was interpreted according to Burnham and Anderson [53]. Although we fitted models using both cell P-quota and biovolume P-quota, we only show results for biovolume quota because the fits were similar and the biovolume quota allows better comparisons between the species. Statistical analyses, curve fitting and model simulations (described below) were performed using R version 3.0.1 [54] with the packages deSolve [55] and FME [56].

### Model and simulations of relative P uptake rates

To estimate the P-uptake rates of *S. minutulus* and *N. acicularis*, we simulated the growth of these species in the competition experiments described above in semi-continuous P-limited culture. The model parameters are described in Table 1. The simulation was run in steps, from one dilution to the next. Between dilutions the two species grew as in a batch culture, such that
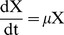
(8)


**Table 1:** Model parameters and variables for simulation of P-competition and relative uptake rates in semi-continuous culture experiments.

Parameter	Meaning	Units
X	Biovolume	mm^3^ L^−1^
Q	P-quota	*µ*g P mm^−3^
S_m_	Concentration of P in fresh medium	*µ*g P L^−1^
V_rel, i_	Biovolume-specific proportion of P absorbed by species i	Dimensionless
*µ*	Specific growth rate, given by Eq. 5 with the parameters in Table 3.	d^−1^
*F*	Dilution factor (proportion of culture retained at dilution)	Dimensionless
T	Time	d
Δt	Time until next dilution	d
D	Dilution rate	d^−1^

Subscripts *i* and *j* refer to the two species.



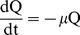
(9)


Because the maximal uptake rate (V_m_) is much higher than required to satisfy immediate needs for growth if cells are substantially P-limited [15], [17], we assumed the P added during dilution was absorbed instantly and completely, so that the dissolved P concentration in the medium was negligible. Furthermore, we assumed that the added P was distributed between the two species (i and j) in fixed proportions, V_rel, i_ and V_rel, j_, such that V_rel, i_+V_rel, j_ = 1. Therefore, at the end of each step the biomass (X) and P-quota (Q) of each species were modified to account for dilution as follows:
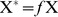
(10)




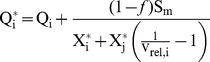
(11)




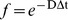
(12)where X* and Q* denote the new biomass and P-quota, respectively, at the start of the next simulation step directly following dilution, S_m_ is the P concentration in the fresh medium, and *f* is the dilution factor, which depends on the dilution rate D and the time until the next dilution, Δt (Eq. 13). Note the subscripts, i and j, which have been introduced because the nutrient absorbed by one species depends on the biomass of the other.

The variables V_rel, i_ and V_rel, j_ are biomass-specific and are therefore proportional to the P-uptake rates of the two species relative to each other. In effect, our approach assumes a linear relationship between external nutrient concentration and uptake rate. The slope of this linear relationship (V_rel, i_ and V_rel, j_) is proportional to the uptake affinity, or the initial slope of the Michaelis-Menten uptake curve V_m_/k_m_, where k_m_ is the half-saturation coefficient [42], [57]. In a calibration routine during the simulations, V_rel, i_ and V_rel, j_ were fitted to the measured data in each competition experiment to investigate how the relative uptake rates change with temperature and photoperiod.

## Results

### P-replete growth

**Figure 1:**
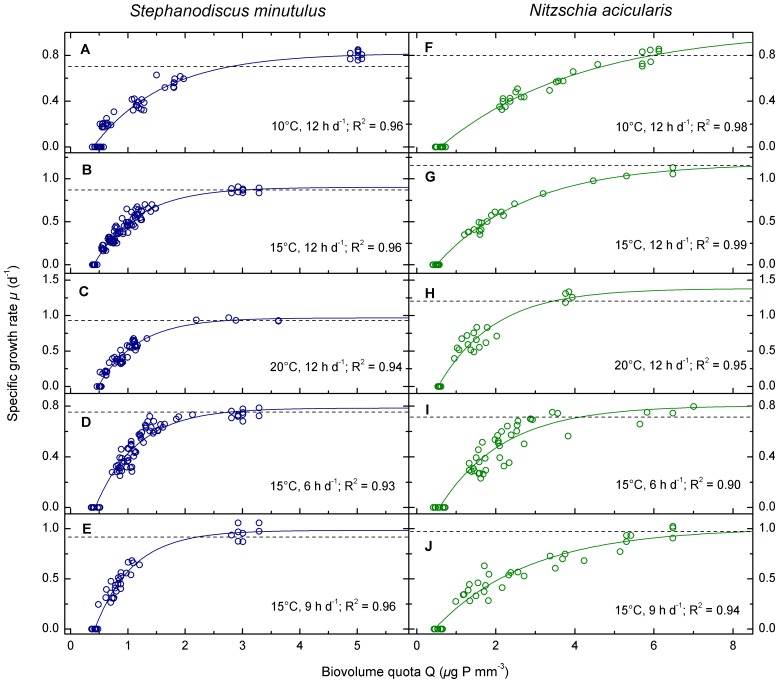
Specific growth rates as a function of biovolume P-quota at different temperatures and photoperiods. Points show measured values for *S. minutulus* (A–E) and *N. acicularis* (F–J), solid lines show the fitted model (Eq. 5) with the parameters in Table 3. The dotted lines show the maximum specific growth rates (*µ*_m_) predicted by the model of Nicklisch *et al.* [11] at the respective temperature, photoperiod and light exposure.

The different models (Eqs. 2–5) were fitted to the growth rates of *N. acicularis* and *S. minutulus* under P-limited and P-replete conditions. Eq. 5 performed the best overall for both species and the Droop model (Eq. 2) performed worst (Table 2). The normalised, three-parameter models (Eqs. 4, 5) performed better for *N. acicularis* whereas the exponential models (Eqs. 3, 5) tended to perform better for *S. minutulus*. Therefore, the normalised exponential model Eq. 5 was used to compare temperature and photoperiod treatments for the following analysis (Figure 1).

**Table 2:** Comparison of different models fitted to experimental results.

Experimental conditions		ΔAIC			
Temperature (°C)	Photoperiod (h d^−1^)	Eq. 2 after Droop [26]	Eq. 3 after Fuhs [49]	Eq. 4 after Flynn [50]	Eq. 5
*Stephanodiscus minutulus*					
10	12	41.4	39.1	0	2.7
15	12	71.4	16.0	7.3	0
20	12	10.7	0	5.2	2.0
15	6	46.2	2.2	15.8	0
15	9	20.7	0	3.3	1.1
*Nitzschia acicularis*					
10	12	80.9	75.3	0.2	0
15	12	91.3	85.3	3.5	0
20	12	19.2	13.3	0	2.7
15	6	36.9	17.4	5.5	0
15	9	44.8	37.0	0	3.9

Lower ΔAIC values indicate better model fits (best model = 0). ΔAIC <2 indicates substantial support that the model in question is the best model; ΔAIC >10 indicates essentially no support [53].

**Figure 2:**
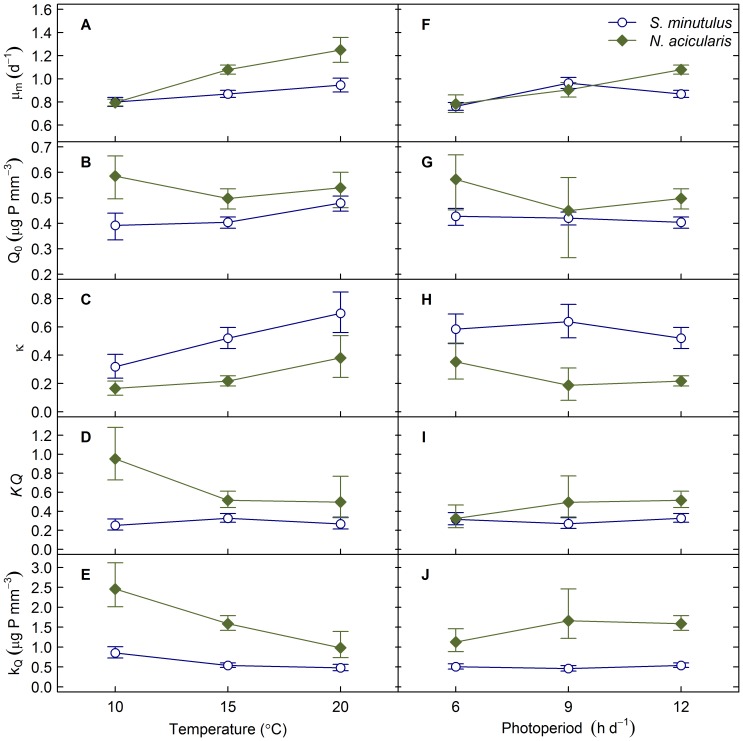
Temperature and photoperiod dependence of physiological growth parameters for *S. minutulus* and *N. acicularis*. The temperature dependence (A–E) was measured at 12 h d^−1^ photoperiod and the photoperiod dependence (F–J) at 15°C. The physiological parameters maximum specific growth rate (*µ*_m_), minimum P-quota (Q_0_), normalised shape parameter (*κ*) and half saturation coefficient (k_Q_) were fitted using Eq. 5, and the normalised shape parameter (*KQ*) was fitted using Eq. 4.

The maximum (P-replete) specific growth rates increased with increasing temperature and photoperiod for both species as expected (Figure 2). *N. acicularis* had a higher maximum specific growth rate (*µ*_m_) than *S. minutulus* at 10–20 °C and 12 h d^−1^ photoperiod but growth rates were similar under shorter photoperiods.

The maximum P-quota (Q_m_) when cells were growing at their maximum rate (*µ*_m_) under luxury consumption decreased with increasing temperature from about 5 *µ*g P mm^−3^ at 10°C to 3 *µ*g P mm^−3^ at 15 °C and 20 °C for *S. minutulus* (p<0.001, Table 3). Overall *N. acicularis* had a higher P-quota than *S. minutulus* (p<0.001), with Q_m_ decreasing significantly (p<0.001) from 6 *µ*g P mm^−3^ at 10 °C to 4 *µ*g P mm^−3^ at 20 °C.

**Table 3:** Fitted model parameters (Eq. 5) for *S. minutulus* and *N. acicularis*.

Experimental conditions		Measured parameters		Fitted parameters (Eq. 5)			
T (°C)	LP (h d^−1^)	Cell size (*µ*m^3^ cell^−1^)	Q_m_ (*µ*g P mm^−3^)	*µ*_m_ (d^−1^)	Q_0_ (*µ*g P mm^−3^)	*κ* (dimensionless)	RSE (d^−1^)
*S. minutulus*							
10	12	185	4.99	0.80 (0.76–0.84)	0.39 (0.34–0.44)	0.32 (0.24–0.41)	0.059
15	12	175	3.01	0.87 (0.84–0.90)	0.40 (0.38–0.43)	0.52 (0.45–0.60)	0.052
20	12	164	3.09	0.95 (0.89–1.01)	0.48 (0.45–0.51)	0.70 (0.56–0.85)	0.066
15	6	184	–	0.76 (0.73–0.80)	0.43 (0.39–0.46)	0.58 (0.48–0.69)	0.063
15	9	189	–	0.96 (0.92–1.01)	0.42 (0.39–0.44)	0.64 (0.52–0.76)	0.068
*N. acicularis*							
10	12	109	5.91	0.80 (0.77–0.82)	0.59 (0.50–0.66)	0.17 (0.12–0.22)	0.039
15	12	107	5.89	1.08 (1.04–1.12)	0.50 (0.46–0.54)	0.22 (0.18–0.25)	0.036
20	12	94	3.85	1.25 (1.14–1.36)	0.54 (0.46–0.60)	0.38 (0.24–0.54)	0.103
15	6	93	6.44	0.78 (0.71–0.86)	0.57 (0.45–0.67)	0.35 (0.23–0.49)	0.087
15	9	99	5.73	0.90 (0.84–0.97)	0.45 (0.26–0.58)	0.19 (0.08–0.31)	0.082

T: temperature, LP: photoperiod, Q_m_: maximum P-quota measured under nutrient replete conditions, *µ*_m_: maximum (nutrient replete) specific growth rate, RSE: residual standard error, 95% confidence intervals are shown in parentheses.

### Temperature and photoperiod effects on P-limited growth

The minimum P-quota required for growth (Q_0_) was lower for *S. minutulus* than for *N. acicularis*, and the normalised shape parameter *κ*, which provides an indication of the efficiency of P usage, was higher for *S. minutulus* (Figure 2C, H). This shows that *S. minutulus* is more efficient and can produce more biomass from a given amount of internal P than *N. acicularis*. Q_0_ was independent of both temperature and photoperiod for both species (p≥0.39, all cases). Although model fits suggested that Q_0_ was significantly higher for *S. minutulus* at 20°C than in the other experiments (Table 3), there was no significant difference between temperature treatments when the quotas measured in cultures grown to stationarity (*µ* = 0) were compared directly by ANOVA (p = 0.43).

The normalised shape parameter *κ* did not change significantly with photoperiod for either *S. minutulus* (p = 0.80) or *N. acicularis* (p = 0.09, Figure 2H). *κ* increased significantly with increasing temperature in *S. minutulus* and *N. acicularis* (p≤0.001 in both cases, Figure 2C). We also checked the temperature and photoperiod dependence of the normalised parameter *KQ* in Eq. 4, which has a somewhat different meaning to *κ* because it includes the temperature dependent Q_m_. *KQ* decreased with increasing temperature (p = 0.03) and increased with photoperiod (p = 0.02) for *N. acicularis* but was constant over temperature and photoperiod for *S. minutulus* (Figure 2D, I). The half saturation coefficient k_Q_, which can be calculated from Eq. 7, overall decreased significantly with increasing temperature in both species (p≤0.001), although in *S. minutulus*, k_Q_ was not significantly different at 15 and 20°C (p = 0.19, Figure 2E). The temperature dependence of k_Q_ was substantially weaker (flatter in Figure 2E) for *S. minutulus* than *N. acicularis*. In *S. minutulus*, *κ* was approximately equal to ln 2 ( = 0.69) (except at 10°C, where *κ* = 0.32), whereas in *N. acicularis κ* was lower than ln 2 by a factor of 2 to 4 (Table 3). Therefore the simpler Droop (Eq. 2) and Fuhs (Eq. 3) models, which are half saturated at 2Q_0_ (i.e. such that *κ* = ln 2), provided a reasonably good description of the growth of *S. minutulus* but not of *N. acicularis*.

### Model of factor interactions

To describe the interactions between temperature, photoperiod and P-limited growth, we used the model of Nicklisch *et al*. [11], which considers the interactions between light exposure, photoperiod and temperature, to calculate the maximum growth rates (*µ*_m_), and coupled it to a normalised quota model to account for P-limitation. The maximum growth rates predicted by the model of Nicklisch *et al*. agreed very well with the maximum growth rates measured under P-replete conditions (dotted line in Figure 1) for both species. For the quota model, the temperature and photoperiod dependencies of the model parameters shown in Figure 2 were formulated in model terms. Because Q_0_ was independent of photoperiod and temperature in both species, it was assumed constant in the quota model formulations. Therefore we tested Eq. 4 with constant *KQ* and Eq. 5 with temperature dependent *κ* as follows:
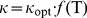
(13)




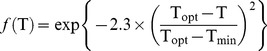
(14)Where *κ*_opt_ is the normalised shape parameter at optimum temperature and *f*(T) is the temperature function after Lehmann *et al.* [58] used in the model of Nicklisch *et al*. [11] with temperature T, optimum temperature T_opt_ and minimum temperature T_min_ (all °C). Fits of both normalised quota models to the whole data set for each species showed that there was very strong evidence (ΔAIC >10) that model 5 with a temperature dependent *κ* (*S. minutulus*: AIC = −822.1, *N. acicularis*: AIC = −358.4) described the measured growth rates of each species better than model 4 with constant *KQ* (*S. minutulus*: AIC = −809.8, *N. acicularis*: AIC = −328.8). This is in agreement with the model comparisons of the individual experiments described above, where model 5 produced the best fit overall.

**Figure 3:**
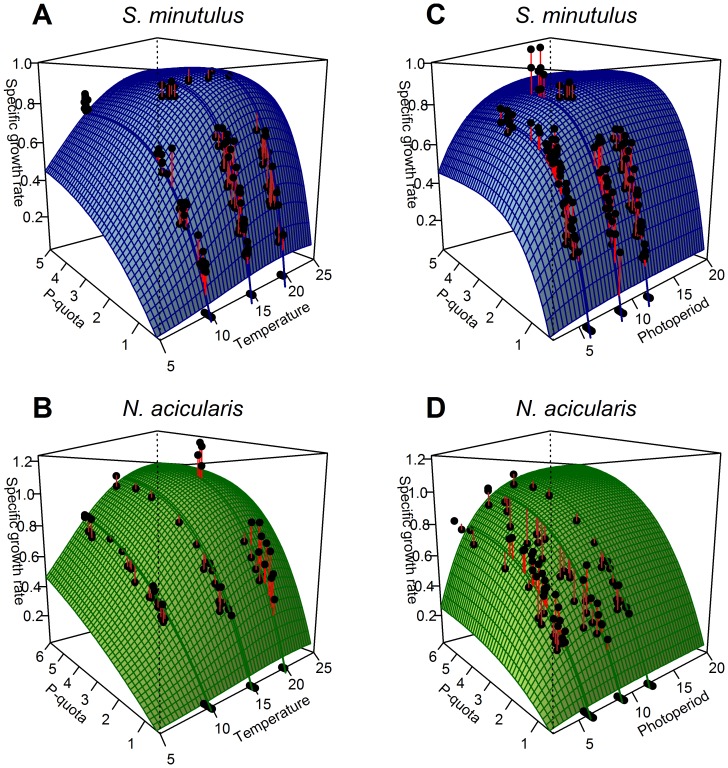
Interaction diagrams showing interactions between P-quota, temperature and photoperiod. Diagrams show the response of specific growth rate (d^−1^) to the combined effect of temperature (°C) and P-quota (*µ*g P mm^−3^) at 12 h d^−1^ photoperiod for *S. minutulus* (A) and *N. acicularis* (B), and also to the combined effect of photoperiod (h d^−1^) and P-quota at 15°C for *S. minutulus* (C) and *N. acicularis* (D).

Therefore we adopted model 5 with the parameters in Table 4, which overall provided a good fit, and used this model to plot the response surfaces under combined limitation by phosphorus and temperature or photoperiod (Figure 3). *S. minutulus* could achieve the same growth rate as *N. acicularis* at considerably lower temperatures, photoperiod and P-quota combinations. A horizontal cross section through the response surface shows growth isoclines (constant growth rate) as in Tilman’s [59] interaction diagrams. The curved regions of these growth isoclines indicate that an increase in photoperiod and temperature can compensate a decrease in P-quota and the regions of interaction correspond to the prevailing spring conditions for these species (e.g. roughly 8–15°C and 4–8 h d^−1^ photoperiods at a growth rate *µ* = 0.5).

**Table 4:** Model of factor interactions (Eqs. 5, 14, 15) and corresponding parameters for P-limited growth of *S. minutulus* and *N. acicularis*.

Parameter	Description	Units	*S. minutulus*	*N. acicularis*
*µ*_m_	Maximum (P-replete) specific growth rate	d^−1^	a	a
Q_0_	Minimum P-quota	*µ*g P mm^−3^	0.43 (0.41–0.44)	0.53 (0.49–0.57)
*κ*_opt_	Normalised shape parameter at optimum temperature	Dimensionless	0.69 (0.64–0.74)	0.34 (0.29–0.39)
T_opt_	Optimum temperature	°C	20.7^a^	21.7^a^
T_min_	Minimum temperature	°C	–0.6^a^	1.0^a^
RSE		d^−1^	0.070	0.092
df		–	330	185

a Calculated or adopted from Nicklisch *et al*. [11].

RSE: residual standard error, df: degrees of freedom, 95% confidence intervals shown in parentheses.

### Competition experiments and relative P uptake rates

In competition experiments under P-limitation, *N. acicularis* was the stronger competitor at all temperatures and photoperiods tested. The experiments showed that, relative to *N. acicularis*, the competitive ability of *S. minutulus* increased with decreasing temperatures under a 12 h d^−1^ photoperiod (Table 5, p<0.001, multiple linear regression on time and temperature) and was higher at 6 h d^−1^ photoperiod than at 12 h d^−1^ photoperiod at 15°C (p<0.001, t-test on linear regression slopes).

**Table 5:** Cultivation conditions and growth rates for P-competition experiments between *N. acicularis* and *S. minutulus*.

T (°C)	LP (h d^−1^)	No. cultures	df	D (d^−1^)	*µ*_net_ (d^−1^)	SE (d^−1^)	*µ* (d^−1^)	CA
15	6	4	40	0.4	−0.127	0.012	0.273	0.683
10	12	4	32	0.4	−0.048	0.003	0.352	0.880
15	12	4	26	0.5	−0.250	0.013	0.250	0.500
20	12	2	10	0.5	−0.298	0.024	0.202	0.404

T: temperature, LP: photoperiod, LE: daily light exposure, I: irradiance, df: degrees of freedom, D: applied dilution rate, *µ*_net_: net growth rate of the weaker competitor (*S. minutulus* in all cases), SE: standard error, *µ*: specific growth rate of the weaker competitor, CA: competitive ability of the weaker competitor (Eq. 1). Light exposures in the experiments were ca. 4 mol m^−2^ d^−1^.

**Figure 4:**
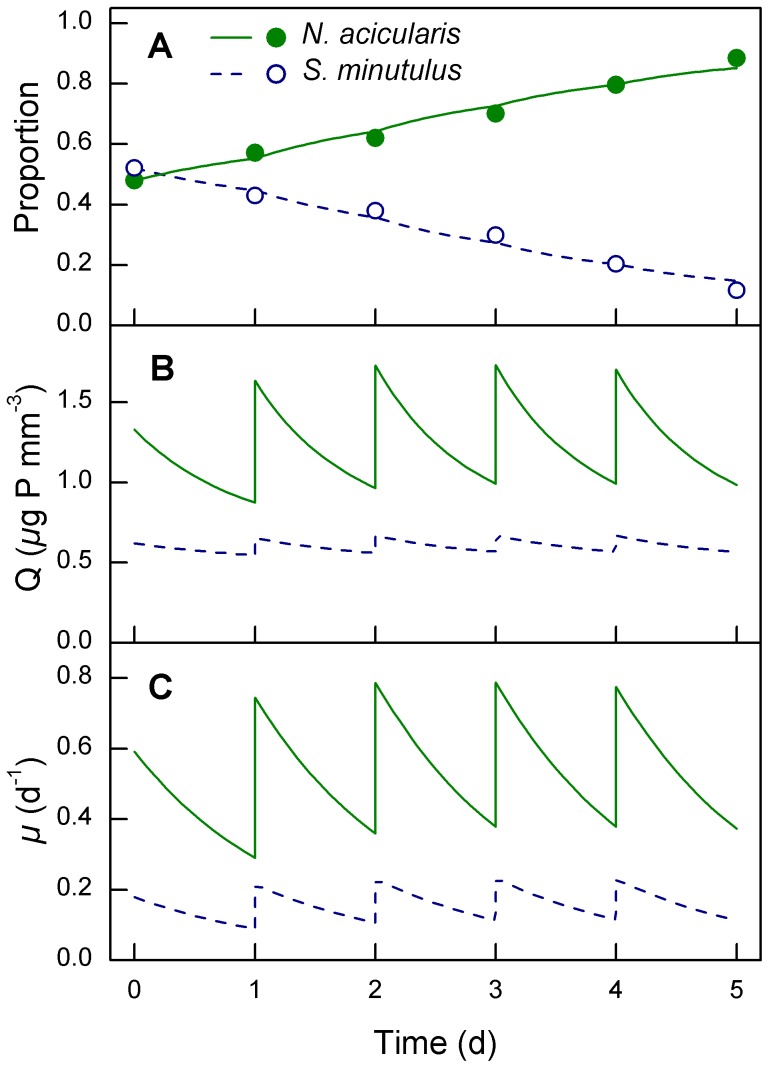
Simulation of P-competition experiments between *N. acicularis* and *S. minutulus.* This example experiment was performed at 15°C, 12 h d^−1^ photoperiod and 3.7 mol PAR m^−2^ d^−1^. Lines show the simulated values of (A) proportions of each species by biovolume, (B) the biovolume P-quotas, and (C) specific growth rates. Points show measured values. *N. acicularis* was the stronger competitor (solid line) and *S. minutulus* the weaker competitor (dashed line).

**Figure 5:**
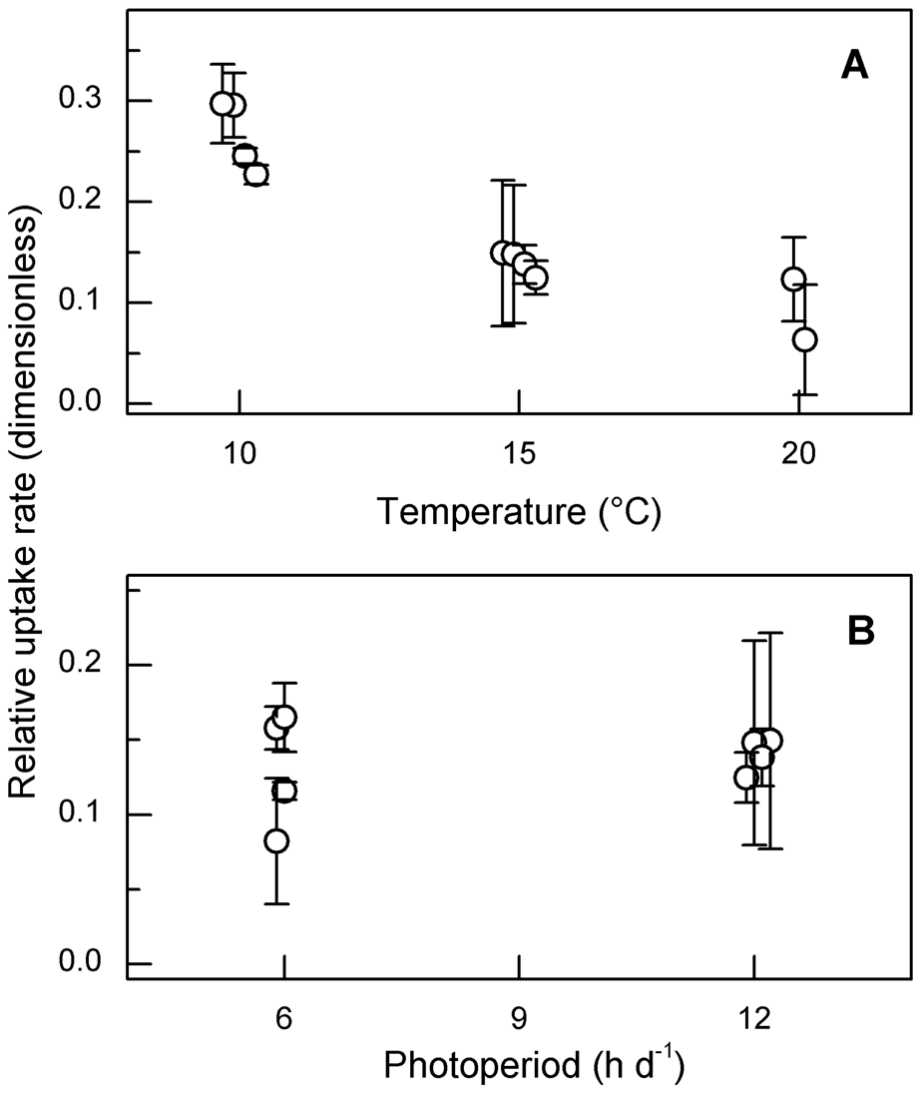
P-uptake rates of *S. minutulus* relative to *N. acicularis*. The relative uptake rates were measured at different temperatures with a 12^−1^ photoperiod (A) and at different photoperiods at 15°C (B). Open circles show the fitted values of parameter V_rel_ for *S. minutulus*, error bars show 95% confidence intervals of the fitted values. The dimensionless relative uptake rates (V_rel_) shown refer to the proportion (%) of added P absorbed by *S. minutulus*. It follows that the proportion absorbed by *N. acicularis* is 1– V_rel_ of *S. minutulus*.

The relative uptake rates were estimated from the model simulations, which accurately described the measured cell proportions of the two species in the competition experiments (Figure 4). The estimated relative uptake rates (Figure 5) showed a similar pattern to the competitive ability results shown in Table 5. The simulations showed that the uptake rate of *S. minutulus* relative to *N. acicularis* was higher at 10°C compared to 15 and 20°C (Table 5, p = 0.0004, ANOVA). At 10°C under a photoperiod of 12 h d^−1^, *S. minutulus* absorbed about 25–30% of the added P, whereas at 20°C it absorbed around 10%, while *N. acicularis* absorbed 90%. There was no significant difference between the relative uptake rates at 6 h d^−1^ and 12 h d^−1^ photoperiod (p = 0.6), where *S. minutulus* and *N. acicularis* absorbed on average 14% and 86% of added P, respectively.

### Carbon content

The mean carbon contents of *N. acicularis* and *S. minutulus* were 148±39 *µ*g C mm^−3^ (n = 15) and 132±18 *µ*g C mm^−3^ (n = 13), respectively. In *S. minutulus*, the carbon content per unit biovolume did not change with dilution rate under P-limitation (p = 0.28). In *N. acicularis* carbon content per unit biovolume increased with increasing dilution rate under P-limitation according to C:X = 118×D+56 (R^2^ = 0.82, p<0.01), where C:X is the carbon: biovolume ratio (*µ*g C mm^−3^) and D is the dilution rate (d^−1^).

### Chlorophyll a content

**Figure 6:**
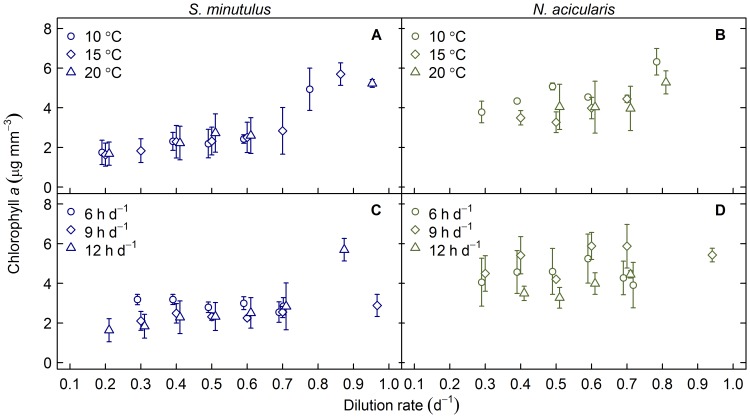
Chlorophyll *a* content at different levels of P limitation (dilution rate). Different symbols show temperature treatments at 12^−1^ photoperiod (A, B) and photoperiod treatments at 15°C (C, D) for *S. minutulus* (left panels) and *N. acicularis* (right panels). Symbols have been staggered horizontally to avoid overlap; dilution rates increased from 0.2 d^−1^ to 0.7 d^−1^ at 0.1 d^−1^ intervals. Higher dilution rates show maximum growth rates. Error bars ±1 SD.

Chlorophyll *a* content increased slightly with increasing dilution rate (decreasing P limitation) up to 0.7 d^−1^ in *S. minutulus* and was relatively constant over this range for *N. acicularis* (Figure 6). Chlorophyll *a* was higher in P replete cultures (dilution rate above 0.7 d^−1^) than in P limited cultures in both species. The mean chlorophyll *a* content of *S. minutulus* and *N. acicularis* under P limitation was 2.38±0.77 *µ*g mm^−3^ (*n* = 195) and 4.33±1.13 *µ*g mm^−3^ (*n* = 115), respectively. Under nutrient replete conditions, the chlorophyll *a* content was 4.59±1.36 *µ*g mm^−3^ (*n* = 28) and 6.28±2.49 *µ*g mm^−3^ (*n* = 31), respectively for the two species. Under P limitation, chlorophyll *a* did not significantly depend on temperature for either species (*p*>0.28) but did depend on photoperiod for both species (*p*<0.001), where the content was higher under short photoperiods than under long photoperiods.

## Discussion

Our study demonstrated that temperature influenced the relationship between growth and P-quota of *S. minutulus* and *N. acicularis*, but the effect of photoperiod length at constant daily light exposure was not significant. Moreover the effect of temperature differed between species in a way that a fixed quota curve like the Droop model could not entirely account for. Similarly temperature also influenced the relative phosphorus uptake rates of the two species but the photoperiod apparently had no influence here. We therefore confirm our hypothesis that there is a complex interaction between temperature and phosphorus limitation but reject our claim that the photoperiod has an effect.

### P-replete growth and model comparison

In our growth experiments, the measured P-replete growth rates (*µ*_m_) increased non-linearly as expected with increasing temperature and photoperiod. These growth rates agreed very well with the predictions of a previously published model [11].

One reason for the Droop model’s poor performance compared to the other tested models is that the fixed form of the Droop equation could not adequately describe the growth rates of *N. acicularis* because *κ*<ln 2. There are other examples where the Droop equation did not apply as evident from non-linear relationships between *µ* and 1/Q [9], [30], [32], [60], [61], which typically occur when the growth-quota curve has a flatter form such that *κ*<ln 2. This explains why the 3-parameter models (Eqs. 4, 5) fitted better than the 2-parameter models (Eqs. 2, 3) for *N. acicularis*. Although the 3-parameter (variable form) curves also provided a significantly better fit for *S. minutulus* than the 2-parameter (fixed form) curves, a 2-parameter model would in our opinion be adequate for this species.

We used biovolume to represent biomass as the basis for P-quota estimates. In diatoms, which can be quite vacuolated, biovolume can lead to biased estimates of the biomass. An alternative is to use C as the quota basis, which is useful as a common biomass currency in modelling studies. However, C-content can also lead to biased estimates of biomass under nutrient limitation when excess photosynthates are stored, such as chrysolaminaran in diatoms [62]. Dry weight poses difficulties in diatoms due to their silicate frustules and cell number can cause problems due to variability of cell size [27]. We chose biovolume because it is fast and easy to measure and it allows modelling estimates of individual species to be verified with field samples, which is not possible with C based quotas. In our experiments, the results were similar using biovolume based and cell based P-quotas, and for *S. minutulus* also C based quotas because C content per unit biovolume was constant. In *N. acicularis*, C per biovolume increased with dilution rate. Using a C based P-quota would therefore result in a more linear quota curve and further decrease *κ* below ln 2.

### Temperature

Our results showed that the P-quota increased with decreasing temperature for a given growth rate as evident from a temperature-dependent *κ* and Q_m_, which is in accordance with other studies [13], [33]. The normalised shape parameter *κ* clearly increased with decreasing temperature in both species, which indicates that the shape of the quota curve changes with temperature. We showed that the temperature dependence of *κ* is approximately the same as the temperature dependence of nutrient replete growth (c.f. Eqs. 14, 15), although it is unlikely to be exactly the same [30]. In absolute terms the increase in k_Q_ with decreasing temperature was only small (but nevertheless significant) for *S. minutulus*. An increase in k_Q_ corresponds to a decrease in growth rate at a certain P-quota, which suggests that the much smaller temperature effect on k_Q_ may reflect an adaptive strategy of *S. minutulus* because it is a cold-adapted, early spring species whereas *N. acicularis* is adapted to warmer temperatures and longer photoperiods and typically grows in late spring [11], [38].

The absence of a temperature dependency of the minimum quota Q_0_ in our study is not consistent with other findings that Q_0_ increases with decreasing temperature [13], [30], [32], although there are some examples where Q_0_ did not increase with decreasing temperature [33], [34], [49]. A decrease in cell size with increasing temperature [13], which we also observed in our study (Table 3), might partially explain this discrepancy if nutrient quotas are given on a cell basis [14], but there does not seem to be a consistent pattern in the literature to provide a clear answer to this. Another explanation could be that the discrepancy is due to methodological differences or curve fitting because the choice of model considerably affects the parameter values. Furthermore, if the Droop equation is fitted, then a change in Q_0_ with temperature will also reflect a change in *κ* or k_Q_ due to the fixed form of the quota curve, so that a temperature dependence of Q_0_ found by model fitting could be just a model artefact. It is therefore interesting to note that studies that found a temperature dependence of Q_0_ estimated this as the axis intercept extrapolated from a fitted curve [13], [30], [32]. On the other hand, the studies that found no temperature dependence measured Q_0_ directly from the maximum yield of cultures grown to stationary phase [33, 34; this study]. One exception was the study of Fuhs [49], who estimated Q_0_ by extrapolation but nevertheless did not find a temperature dependence. Ahlgren [14] also questioned the significance of the temperature dependence of Q_0_ for phosphorus in some studies. We checked the possibility that the temperature dependence of Q_0_ could be an artefact of model fitting by removing the data points measured at stationarity (*µ* = 0) and refitting the Droop equation to our data. This resulted in a highly significant temperature dependence of Q_0_ for *N. acicularis* but no temperature dependence for *S. minutulus*. This is consistent with the need for additional P-rich ribosomes [63] to compensate a decrease in temperature and still maintain the same protein synthesis and growth rate [13], [47], [64]. However, this may not apply when the growth rate is zero. We suggest that further research is necessary to clarify whether Q_0_ is temperature dependent because this is relevant for optimum N:P ratios and overall phytoplankton stoichiometry [65].

The majority of published results (e.g. [13], [32]) and our results for *N. acicularis* indicate that the form of the quota curve changes with temperature, making a fixed-form curve such as the Droop model inappropriate in most cases to account for temperature interactions with phosphorus [30]. The fact that the Droop relation applied reasonably well for *S. minutulus* and the temperature interactions with phosphorus-limited growth could be relatively well described by a multiplicative model with a fixed curve is presumably due to specific adaptation of this cold-water species and serves to highlight the species-specific nature of the effect of temperature on P-limited growth.

### Photoperiod

**Figure 7:**
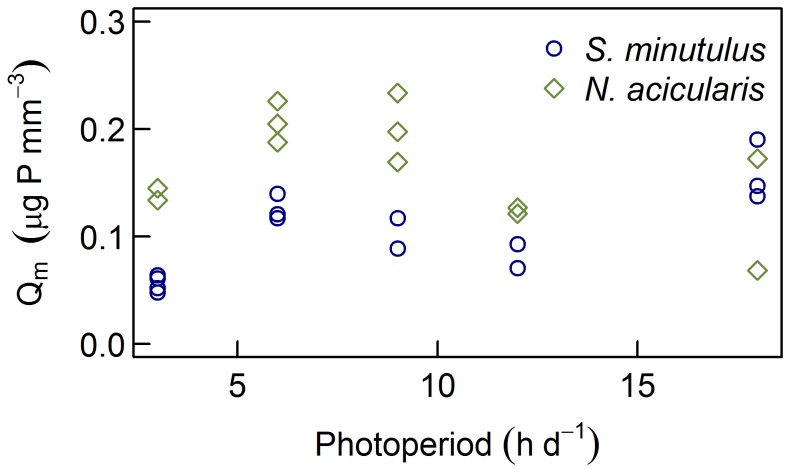
Maximum P-quotas (Q_m_) at different photoperiods. The data shown are for *Stephanodiscus minutulus* and *Nitzschia acicularis* measured at 20°C in semi-continuous culture in P-replete medium, and were published in Giersdorf [66].

The lack of a significant effect of the photoperiod on Q_0_ or *κ* indicates that the photoperiod does not alter the shape of the quota curve for *S. minutulus* or *N. acicularis*. Further, it suggests that the effects of photoperiod and P-limitation are multiplicative and that a decrease in the photoperiod would require an increase in P-quota to maintain the same growth rate, as was evident from the interaction plots (Figure 3). This is consistent with the results of Litchman *et al*. [20], who found that the response of several phytoplankton species to daylength depended on the P-status and vice versa. A similar result was also found for *Limnothrix* (formerly *Oscillatoria*) *redekei* because k_Q_ and Q_0_ were the same under both continuous light and a photoperiod of 12 h d^−1^ [33]. On the other hand, Riegman and Mur [21] found only a narrow range of interaction between internal phosphorus and the photoperiod for *Planktothrix agardhii*. These authors suggested that Liebig’s Minimum Law applies to *P. agardhii* because it is a shade adapted species and they hypothesised that high light adapted species might have a broader interaction range. We used our model, which contains a multiplicative interaction between P-quota and photoperiod, to examine the interactions of *S. minutulus* and *N. acicularis* (which are slightly less shade-adapted than *P. agardhii*) under the same conditions as used by Riegman and Mur (results not shown). The interaction range was somewhat broader, supporting Riegman and Mur’s hypothesis, but also demonstrating that a multiplicative interaction can produce visually similar results at the low growth rates these authors used (0.01 h^−1^). Evidence shows that a decrease in irradiance (*µ*mol photons m^−2^ s^−1^) generally requires an increase in P-quota to maintain the same growth rate, suggesting an interaction between light and P-limitation [9], [14]. Furthermore, Healey’s [9] results demonstrated that Q_0_ and Q_m_ for phosphorus (both cell- and C-based quotas) were independent of irradiance, indicating that the interaction between irradiance and phosphorus is multiplicative. This might help to explain the similar interaction with the photoperiod in our study, where k_Q_ and *κ* were independent of the photoperiod and, according to Healey’s results, also the irradiance. Further evidence is provided by the fact that Q_m_ in *S. minutulus* and *N. acicularis* is unaffected by either irradiance or photoperiod (Figure 7) [66].

We found that chlorophyll content decreased under P limitation, which is consistent with other studies [31], [67], [68]. Since chlorophyll increases under light limiting conditions, photoacclimation as well as light-phosphorus interactions can play a role under combined nutrient and light limitation. This should be more relevant in eutrophic lakes, which through self-shading tend to be more turbid with a shallower euphotic depth and shorter effective photoperiod than in clear, strongly P limited oligotrophic lakes. Therefore, the type of interaction between phosphorus and photoperiod or light should be accounted for when coupling a quota model to a model of light limitation. We do not expect that photoacclimation influenced our results because the cultures were grown at steady state.

### Competition and P-uptake

To estimate relative P uptake rates, we employed a new approach which uses the relationship between growth and P-quota and the outcome of competition experiments. This approach allowed us to quantify the contribution of P-uptake and efficiency of P-usage to competitive ability, but it could not produce absolute values of uptake rates. Our assumption of a simple linear relationship between uptake rate and external concentration can provide reliable results [69], [70] and applies if the external P concentration is lower than the half-saturation coefficient of uptake (k_m_). The P-concentration in our fresh medium (1.2 *µ*M P) was lower than measured k_m_ values for most species provided by Gotham and Rhee [71] (mean 1.5 *µ*M P, n = 6) and the external concentrations in our cultures can therefore generally be expected to be much lower than k_m_. Our linear relationship also ignores the feedback of the quota, which causes V_m_ to decrease as Q increases. However, the P-quota was always lower than Q_m_ in model simulations for both species, except very occasionally after cultures were strongly diluted. Furthermore, this feedback depends on nutritional history rather than the instantaneous P-status [69], [72] allowing phytoplankton to overshoot their maximal quotas in the short term [17]. Therefore, we assume that ignoring the feedback from the quota did not affect our results to any great degree.

The outcome of the experiments showed that *N. acicularis* was a strong competitor under P-limitation, which is consistent with other studies [43], [73]. Interestingly *S. minutulus*, which was the weaker competitor under all experimental conditions, could use internal P more efficiently than *N. acicularis* under most conditions, due to its lower Q_0_ and higher *κ*. The competitive advantage of *N. acicularis* was due to a higher uptake affinity. Other studies found similar results, where the stronger of two species competing under nutrient limitation had higher uptake rates and a higher Q_m_ whereas the weaker competitor produced a higher yield from a given amount of nutrient [69], [74]. A high maximum quota in combination with a high uptake rate enables a species to maintain a relatively high quota so that efficient use of internal P for growth is less important. This may suggest a trade-off between resource gathering and resource usage in biomass assembly [28] and stresses the importance of linking uptake and the quota curve [27]. It thus seems that the parameters of the quota curve alone provide little information on the competitive ability of a species without knowledge of the uptake kinetics. For this reason, Flynn [50] concluded that, in addition to the growth-quota relationship, models need to account for surge nutrient transport to properly describe competition between species.

The influence of temperature on relative P-uptake rates was clear in the competition experiments. It was not possible to estimate the absolute temperature dependencies of uptake for each species, but it was possible to deduce how P was distributed between the species and how temperature and photoperiod influenced this distribution. *S. minutulus* increased its share of absorbed P from around 10% at 20°C to 25–30% at 10°C, indicating that the uptake kinetics of *S. minutulus* are more cold-adapted than those of *N. acicularis*, which parallels the interaction between temperature and the growth-quota curve described above.

The photoperiod appeared to have no influence on the relative uptake rates of the two test species. We cannot rule out that the photoperiod did affect the absolute uptake rates of each species but that these effects cancelled each other out; however, we assume this is unlikely because there was no evidence that the photoperiod influenced other P-limited growth parameters like *κ*, k_Q_ or Q_0_. Sommer [75] found that the total daily light exposure rather than photoperiod or irradiance alone influenced the outcome of competition between marine phytoplankton under Si or N limitation. In our competition experiments, we tried to keep the daily light exposure constant but still found that the photoperiod influenced the outcome. However, this was obviously due to the effect of the photoperiod on maximum growth rates because relative P-uptake rates were unaffected.

Altogether our study showed that temperature interactions with P-limited growth are complex and reflect species-specific niche adaptation. The influence of the photoperiod seems to be restricted to nutrient replete rather than P-limited growth rates, although the same need not apply to N-limitation. The nature of the temperature interactions with P-quota suggests that warming should counteract de-eutrophication and, when phosphorus is limiting, favour warm-adapted species more than expected based only on their temperature optima.
