# Correction: Temperature and Photoperiod Interactions with Phosphorus-Limited Growth and Competition of Two Diatoms

**DOI:** 10.1371/journal.pone.0111869

**Published:** 2014-10-21

**Authors:** The PLOS ONE Staff

There is an error in the citation for Equation 5. The citation is: Fuhs [49].

In Table 1, the parameters for dilution factor and time are incorrect. The parameter for dilution factor should be “*f*”, and the parameter for time should be “t”. Please see the corrected Table 1 here.

**Table 1:** Model parameters and variables for simulation of P-competition and relative uptake rates in semi-continuous culture experiments.

Parameter	Meaning	Units
X	Biovolume	mm^3^ L^-1^
Q	P-quota	*µ*g P mm^-3^
S_m_	Concentration of P in fresh medium	*µ*g P L^-1^
V_rel, i_	Biovolume-specific proportion of P absorbed by species i	Dimensionless
*µ*	Specific growth rate, given by Eq. 5 with the parameters in Table 3.	d^-1^
*f*	Dilution factor (proportion of culture retained at dilution)	Dimensionless
t	Time	d
Δt	Time until next dilution	d
D	Dilution rate	d^-1^

Subscripts *i* and *j* refer to the two species.
